# Successful conservative management of a spontaneous intraperitoneal rupture of bladder diverticulum in a critical patient: A case report. A CARE-compliant article

**DOI:** 10.1097/MD.0000000000019262

**Published:** 2020-02-14

**Authors:** Seong Beom Oh, Jung Hwan Ahn

**Affiliations:** a Department of Emergency Medicine, Dankook University School of Medicine, Cheonan; b Department of Emergency Medicine, Ajou University School of Medicine, Suwon, Republic of Korea.

**Keywords:** conservative treatment, diverticulum, spontaneous rupture, urinary bladder

## Abstract

**Rationale:**

A spontaneous rupture of the bladder diverticulum in an adult patient is extremely rare. The recommended treatment is surgery. However, some cases can be successfully treated with urinary catheterization, antibiotics, and/or percutaneous peritoneal drainage. In this case report, a spontaneous rupture of the bladder diverticulum was successfully treated non-surgically because it was deemed too risky for surgical intervention, such as non-ST segment elevation myocardial infarction (NSTEMI).

**Patient concerns:**

A 76-year-old man presented with abdominal pain, distention, diarrhea, and oliguria for 3 days and hypotension for 1 day with no history of trauma. The patient showed direct and rebound tenderness in the lower abdomen. Computed tomography revealed an intraperitoneal bladder rupture associated with the bladder diverticula. Electrocardiography, echocardiography, and elevated cardiac enzyme suggested NSTEMI.

**Diagnoses:**

A spontaneous rupture of the bladder diverticulum, NSTEMI, and suspected sepsis due to gastroenteritis or urinary infection.

**Interventions:**

The patient was treated conservatively with urinary catheterization and antibiotics for a bladder rupture and an infection. Percutaneous transluminal coronary angioplasty was performed for NSTEMI.

**Outcomes:**

The patient fully recovered without complications on hospitalization day 13.

**Lessons:**

Conservative management might be an alternative for a spontaneous intraperitoneal bladder rupture in some cases. However, close observation is required, and surgical intervention is the first option for a spontaneous intraperitoneal rupture of the bladder diverticulum.

## 1 Introduction

Spontaneous rupture of a bladder diverticulum in adult patient is an extremely rare disease entity. To the best of our best knowledge, only 15 cases have been reported thus far.^[1,2]^ A patient with such condition may experience abdominal pain, abdominal distension, decreased urine output, and other urinary symptoms. The rarity of this disease entity and its non-specific symptoms may result in a delay in accurate diagnosis of the ailment and lead to life-threatening condition.^[1–5]^

Treatment of traumatic bladder rupture is determined by the type of bladder rupture.^[6]^ Bladder contusion, interstitial bladder injury, and extraperitoneal bladder rupture are recommended to be treated by conservative management while intraperitoneal bladder rupture are to be treated by surgery.^[6]^ Surgical treatment is recommended when spontaneous rupture of a bladder diverticulum is an intraperitoneal type such as a traumatic intraperitoneal bladder rupture.^[1,2,4–8]^ However, there are documented cases of spontaneous rupture of bladder diverticulum cured by conservative treatments without surgery.^[3,9–11]^ These case reports suggest that conservative treatment such as urinary catheterization and antibiotics might be an alternative and acceptable treatment option for spontaneous rupture of bladder diverticulum.

This case report describes successful treatment of a patient non-surgically by conservative treatments for a spontaneous intraperitoneal rupture of bladder diverticulum demonstrating an overall too risky condition for surgical intervention.

## 2 Case report

A 76-year-old man with a past history of diabetics, hypertension, benign prostate hypertrophy, and no previous operation history visited the emergency department with complaints of abdominal pain for 3 days and hypotension. Abdominal computed tomography (CT) was performed initially at another hospital to identify causes of the abdominal pain and hypotension (blood pressure, 76/43 mmHg; heart rate, 140/min). He was recommended to visit a higher-level hospital because of sustained hypotension despite treatment. Thus, he was transferred to our emergency room.

**Figure 1:**
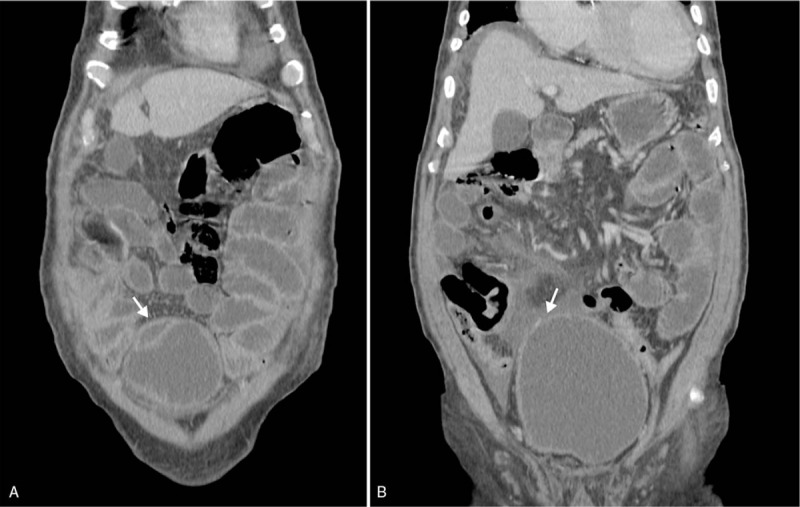
Coronal view of abdominal computed tomography. A. The black arrow indicates suspected infected diverticulum. B. The black arrow shows small defect in the dome of the bladder diverticulum. Small amounts of intraperitoneal fluid were observed.

**Figure 2:**
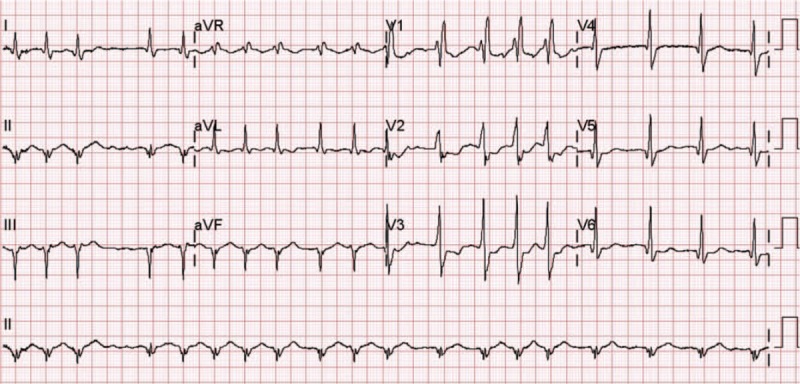
Electrocardiography with right bundle branch block and atrial fibrillation with rapid ventricular response. There is no suspicious ST segment elevation.

Vital signs upon arrival at our hospital were as follows: blood pressure, 121/67 mmHg; heart rate, 134/min; respiration rate, 28/min; and body temperature, 36.7 °C. Detailed history taking revealed that lower abdominal pain arose 3 days ago immediately after 5 times of diarrhea. The patient showed tenderness and rebound tenderness on lower abdomen. CT scan performed at another hospital demonstrated both hydroureteronephrosis, distention of urinary bladder with diverticulum, enlarged prostate gland, and small amount of fluid collection around the bladder with suspicious bladder wall defect in bladder dome (Fig. 1). The patient's symptoms strongly suggested peritonitis from a ruptured bladder. As a result, emergency physicians and the urologist planned to surgically repair the ruptured bladder diverticulum. However, an electrocardiograph (ECG) showed ST segment depression in V2 and V3 that might increase the risk of an acute coronary event. Furthermore, a blood examination revealed elevated cardiac enzyme levels and an echocardiography indicated regional wall motion abnormality in concurrence with results from ECG findings. In ECG, rhythm showed atrial fibrillation with rapid ventricular response and right bundle branch block pattern (pathologic Q wave in II, aVF. ST depression in V2–V3, Fig. 2). In echocardiography, mild dysfunction of left ventricle was seen with ejection fraction of 45%. Regional wall motion abnormalities were seen in inferior, inferolateral walls from base to mid and anterolateral from mid to apex of left ventricle. An emergency coronary angiography was warranted followed by an echocardiography and ECG. The coronary angiography showed chronic occlusion in right coronary artery and 95% stenosis in the left circumflex artery. Percutaneous transluminal coronary angioplasty was done. The patient was quickly transferred to the intensive care unit to concisely manage non-ST segment elevation myocardial infarction (NSTEMI). Laboratory test results were: white blood cells, 15.7 × 10^3^/μL; hemoglobin, 8.0 g/dL; platelet, 138 × 10^3^/μL; C-reactive protein (CRP), 19.4 mg/dL; Creatinine, 3.45 mg/dL; CK-MB, 6.55 ng/mL; Troponin-T, 0.34 ng/mL; and procalcitonin, 2.66 ng/mL. In urine analysis, micro-red blood cell count was 30 to 99/HPF with white blood cell >100/HPF.

With these findings, a decision was made to pursue conservative management instead of immediate surgery for the bladder rupture. After urethral catheterization and antibiotics, the patient experienced decreased lower abdominal pain in several hours. The patient's abdominal pain almost subsided on the second day of admission. Creatinine and CRP levels were decreased to 1.32 and 14 mg/dL, respectively. Blood and sputum culture revealed no growth of microorganism. However, urine culture revealed the growth of *Streptococcus agalactiae*. On the fourth day of admission, rebound tenderness was completely absent. Creatinine and CRP levels were decreased even further to 0.97 and 8.5 mg/dL, respectively. On the 13 day of admission, the patient showed a much improved overall condition without any symptoms related to the bladder rupture. He was discharged from the hospital without complication. At the discharge, laboratory findings were: procalcitonin, 0.06 ng/mL; creatinine, 0.94 mg/dL; CRP, 2.27 mg/dL; white blood cell count, 9140 × 10^3^/μL; platelet, 183 × 10^3^/μL, and Troponin-T, 0.06 ng/mL.

A non-contrast abdomen CT at 3 months after discharge revealed distension of bladder with hydroureteronephrosis. There was no urinary outlet obstruction, peritoneal fluid, bladder diverticula, or rupture of bladder diverticula. The patient appeared to be in full recovery without complications. Patient has provided informed consent for publication of the case report. The approval (IRB no. 2019-05-003) of the Institutional Review Board of Dankook University School of Medicine, Cheonan, Republic of Korea was obtained.

## 3 Discussion

Spontaneous bladder rupture is very rare disease with reported incidence of 1:126,000.^[8,12,13]^ It has been reported to be associated with malignancy and post-radiotherapy,^[14,15]^ after a binge of alcohol or alcohol intoxication,^[8,16]^ candida cystitis,^[17]^ eosinophilic cystitis,^[8]^ and diverticulum.^[1–3,5,7,18]^ Symptoms or signs of the spontaneous bladder rupture are non-specific.^[1–3,5,7,18]^ Therefore, the diagnosis is not made easily.^[1–3,5,7,18]^ Rupture of bladder diverticulum is extremely rare, making it more difficult to diagnosis the bladder rupture.^[1–3,5,7,18]^ Although it is difficult for clinicians to diagnosis spontaneous bladder rupture because of its rarity, suspicious situation with abdominal pain, distention, and voiding difficulty might be helpful to make a diagnosis. Patients diagnosed with spontaneous bladder rupture may exhibit symptoms or signs of full filled bladder such as already having bladder outlet obstruction (bladder malignancy, benign prostatic hyperplasia, urethral stricture) and/or weakened bladder wall (tuberculosis, chronic cystitis, neurogenic bladder, diverticula).^[1–3,5,7,18]^ In addition to these situation, if patients have suspicious situations including elevation of intra-abdominal pressure such as diarrhea or after binge drinking, these clinical clues would be very helpful to diagnose bladder rupture. In the present case, there was no definitive trauma history. We assumed that escalation of intra-abdominal pressure such as diarrhea might have induced the rupture of bladder diverticula which might be associated with chronic cystitis under bladder outlet obstruction (benign prostatic hyperplasia).

Treatment of both traumatic or spontaneous bladder rupture can be made according to anatomical location of bladder rupture.^[1–12,14–20]^ As mentioned above, intraperitoneal type of bladder rupture is recommended to be treated via surgery because it might lead to life-threatening condition.^[1–5]^ A spontaneous intraperitoneal bladder rupture in a healthy patient has been successfully treated by urinary catheterization and antibiotics.^[9]^ Patients diagnosed with intraperitoneal bladder rupture experiencing blunt trauma to the bladder or complications of transurethral resection of bladder tumors have also been treated successfully with conservative management treatments without surgery.^[10,11,14,15,20,21]^ Moreover, successful management of suspected bladder rupture after augmentation enterocystoplasty with only conservative management without surgery has been observed at 87%.^[19]^

Our patient had been diagnosed with NSTEMI. We also observed indicators probably pointing to septic condition. As a result, the patient could only be treated by urethral catheterization and antibiotics. Despite the condition of the patient and the associated risks, the patient was successfully treated conservative management treatments without surgery. It is our understanding that this case report could be one of the first few documented cases of spontaneous intraperitoneal rupture of bladder diverticula successfully treated with conservative management alone in a patient suspected of having septic condition and demonstrating unstable or other high risk symptoms.

These findings suggest that conservative management may be a valid treatment method for spontaneous intraperitoneal bladder rupture, resulting in successful treatment and recovery. Further research is needed to determine the indication for intraperitoneal type of bladder rupture in order to determine if conservative management alone is the most suitable and the best option for the patient.

## Author contributions

**Conceptualization:** Seong Beom Oh, Jung Hwan Ahn.

**Data curation:** Seong Beom Oh, Jung Hwan Ahn.

**Investigation:** Seong Beom Oh, Jung Hwan Ahn.

**Methodology:** Seong Beom Oh.

**Supervision:** Jung Hwan Ahn.

**Writing – original draft:** Seong Beom Oh, Jung Hwan Ahn.

**Writing – review & editing:** Jung Hwan Ahn.
